# The Association Between Hispanic Ethnicity, Cholecystectomy, and Postoperative Mortality

**DOI:** 10.1097/AS9.0000000000000709

**Published:** 2026-09-10

**Authors:** Priyanka Singh, Noah Z. Freundlich, Gezzer Ortega, Robert Parody, Mayur Narayan, Joseph Hanna, Brian L. Strom, Gregory L. Peck

**Affiliations:** From the * Department of Medicine, Icahn School of Medicine at Mount Sinai, New York, NY; † Department of Surgery, Rutgers Robert Wood Johnson Medical School, Piscataway, NJ; ‡ Department of Surgery, Center for Surgery and Public Health, Brigham & Women’s Hospital, Harvard Medical School, Boston, MA; § Department of Mathematics and Statistics, Rochester Institute of Technology College of Science, Rochester, NY; ∥ Department of Surgery, Rutgers Robert Wood Johnson Medical School, New Brunswick, NJ; ¶ Rutgers Institute for Health, Health Care Policy, and Aging Research - Center for Pharmacoepidemiology, New Brunswick, NJ; # Department of Health Behavior, Society, and Policy, Rutgers School of Public Health, Piscataway, NJ.

**Keywords:** Hispanic, ethnicity, cholecystectomy, mortality, gallstone, gallbladder, outcomes, surgery, emergency, elective, outpatient

## Abstract

**Introduction:**

Hispanic ethnicity is associated with an increased incidence of gallstone disease. Emergency cholecystectomy has a higher case fatality rate than nonemergency cholecystectomy, yet it remains common. We aimed to determine whether an association exists between Hispanic ethnicity, cholecystectomy type, and mortality.

**Methods:**

The National Surgical Quality Improvement Program database was queried for cholecystectomy cases from 2005 to 2019. Cases included cholecystectomy type. Primary exposure was Hispanic ethnicity. We compared emergency to outpatient cholecystectomy cases, and unplanned (emergency or urgent) to planned (inpatient elective or outpatient) cholecystectomy cases in Hispanic versus non-Hispanic patients. We then compared mortality for Hispanic to non-Hispanic patients for unplanned and planned cholecystectomies separately.

**Results:**

In total, 479,543 patients underwent cholecystectomy. Hispanic versus non-Hispanic patients had increased odds of emergency compared to outpatient cholecystectomy (adjusted odds ratio [aOR] 2.32; confidence interval [CI]: 2.26, 2.39). Hispanic patients also had increased odds of unplanned cholecystectomies compared with planned cholecystectomies (aOR: 1.59; CI: 1.56, 1.63). Hispanic patients who underwent unplanned cholecystectomies had a 79% decreased odds of mortality compared to non-Hispanic patients (aOR: 0.21; CI: 0.19, 0.23). Hispanic patients who had planned cholecystectomies had a 65% decreased odds of mortality (aOR: 0.35; CI: 0.33, 0.38) compared with non-Hispanic patients.

**Conclusion:**

Hispanic patients are more likely than non-Hispanic patients to undergo emergency versus outpatient and unplanned versus planned cholecystectomy. However, Hispanic patients have lower 30-day postoperative mortality after both unplanned and planned cholecystectomies compared to non-Hispanic patients.

## INTRODUCTION

Cholecystectomy for symptomatic gallstone disease is the most common abdominal surgical procedure.^1,2^ Most cholecystectomies in the acute care setting treat acute cholecystitis, though cholecystectomy is also used to treat biliary pathologies such as choledocholithiasis and gallstone pancreatitis after endoscopic decompression.^3–5^ Hispanic ethnicity has been associated with an increased incidence of gallstone disease.^6^ Many of these patients have an acute-on-chronic presentation and a potential to intervene earlier in the gallstone disease course and prevent the need for higher-risk urgent or emergent surgery.^3,7^ These unplanned surgeries have worse outcomes than those completed in the elective setting.^8^ Although mortality from cholecystectomy is thought to be very low compared to other emergency general surgeries, previous work demonstrates that the case fatality rate of emergency cholecystectomy is as high as three hundred times that of ambulatory cholecystectomy.^9,10^

Disparities in racial and ethnic minority patients undergoing general surgical procedures have been well documented in the literature.^11–15^ Janeway et al^16^ showed that Hispanic patients had decreased odds of undergoing ambulatory versus inpatient cholecystectomy compared with White patients in a multiple-state study. Hall et al^17^ published a national study that demonstrated Black patients have higher odds of dying after emergency general surgery than White patients.

We are not aware of a US population study that has examined the association between Hispanic ethnicity, odds of potentially higher-risk types of cholecystectomy, and mortality. This study aims to determine whether Hispanic patients with symptomatic gallstone disease more frequently undergo emergency cholecystectomy than non-Hispanic patients. We will also determine Hispanic patients’ particular experiences with other types of cholecystectomies not performed as an emergency. We hypothesize that Hispanic patients are more likely than non-Hispanic patients to have emergency rather than outpatient cholecystectomy, undergo more acute forms of other cholecystectomies (e.g., urgent), and thus are more likely to have an increased 30-day postoperative mortality.

## METHODS

### Data Source

This population case–control study of the association between cholecystectomy type and ethnicity and cohort study of the association between cholecystectomy type and mortality used the American College of Surgeons National Surgical Quality Improvement Program (NSQIP) dataset.^18^ The NSQIP dataset is a national database that includes demographic and 30-day postoperative morbidity and mortality data for surgical patients in both inpatient and outpatient settings from over 700 out of a total of 6120 hospitals in 49 out of 50 states in the United States.^19^ NSQIP uses case sampling to collect data from academic, community, teaching, and nonteaching hospitals, and is representative of surgical outcomes at the population level.^20,21^

### Study Time and Population

The population for our study was all individuals in the nation included in the NSQIP database from 2005 to 2019. We selected all cholecystectomy cases from 2005 to 2019 using the Current Procedural Terminology codes (Supplementary Appendix 1, https://links.lww.com/AOSO/A677). We included patients aged 18 years and older who had a gallstone disease diagnosis determined by ICD-9 and ICD-10 codes, and excluded patients who underwent cholecystectomy for diagnoses other than gallstone disease (Supplementary Appendix 1, https://links.lww.com/AOSO/A677). Patients’ demographic characteristics, including age, sex, ethnicity, race, and clinical factors associated with gallstone disease, including smoking, diabetes mellitus, body mass index, recent weight loss, and American Society of Anesthesiologists status, were collected.^3,22–26^

### Study Design and Measures

The primary study is a population case–control study. We identified within the NSQIP population one primary case group: those who had surgical gallstone disease treated with an emergency cholecystectomy, and three control groups of patients who had surgical gallstone disease treated with urgent cholecystectomy, inpatient elective cholecystectomy, and outpatient cholecystectomy. We defined the type of cholecystectomy using NSQIP registrar-collected variables: emergency status, inpatient status, and elective status, which yielded four types: (1) emergency, (2) urgent (i.e., nonemergency-inpatient-nonelective), (3) inpatient elective (i.e., nonemergency-inpatient-elective), and (4) outpatient (i.e., nonemergency outpatient). Our study then evaluated Hispanic versus non-Hispanic ethnicity as exposure. Hispanic ethnicity, a nonmodifiable risk factor, included responses of “yes,” “no,” and “unknown” in the NSQIP dataset. Further, in a second cohort study, our exposures were these four different treatment types, and we assessed 30-day mortality within the same NSQIP dataset, looking at whether the odds of mortality varied by ethnicity.

### Analysis of Hispanic Patients and Emergency Compared With Other Cholecystectomy

Descriptive statistics of patient demographics and clinical factors were calculated using frequencies. The odds of emergency cholecystectomy and each other type of cholecystectomy in Hispanic and non-Hispanic patients were determined. We calculated the crude odds ratio (OR) of the likelihood of emergency compared with outpatient cholecystectomy in Hispanic versus non-Hispanic patients. We then used multivariable logistic regression to calculate the adjusted OR with a 95% confidence interval (CI). Our model was adjusted for age, sex, race, and other potential confounders including smoking, diabetes mellitus, body mass index, recent weight loss, and American Society of Anesthesiologists status.

### Subanalysis of Hispanic Ethnicity and Types of Cholecystectomies

The same approach was used to calculate the adjusted odds of unplanned and planned cholecystectomies in Hispanic and non-Hispanic patients. We included (1) emergency and (2) urgent types in unplanned cholecystectomies and (3) inpatient elective and (4) planned outpatient cholecystectomy in planned (Figure 2).

To test our hypothesis of a disparity for Hispanic patients in need of care for symptomatic gallstone disease no matter the type of cholecystectomy, a polynomial ordinal regression was then used to calculate the cumulative adjusted OR across the four types of cholecystectomies using an a priori least ideal to most ideal framework of patient-centeredness; that is, patients would prefer that if they were to develop gallstone disease, it would have the lowest likelihood of becoming an emergency situation requiring surgery.^27^ Hispanic patients were compared to non-Hispanic patients for the least ideal versus most ideal, that is, (1) emergency versus (4) outpatient; (1) emergency versus (2, 3, 4) nonemergency; (1, 2) unplanned versus (3, 4) planned; and (1, 2, 3) inpatient versus (4) outpatient cholecystectomy. 95% CIs were calculated.

### Analysis of Mortality

We then calculated an adjusted OR with 95% CIs of mortality for Hispanic versus non-Hispanic patients for unplanned surgeries and then separately for planned surgeries. We have included open versus laparoscopic surgery as a potential confounder in the multivariate model.

### Sensitivity Analyses of Type of Cholecystectomies and Mortality

We then assessed the impact on the association between Hispanic ethnicity and type of cholecystectomy by including 93,387 patients (18.5%) who were not included in our primary analysis because of “unknown” ethnicity or missing ethnicity. First, we included “unknown” or missing in the Hispanic group to examine bias. Next, we assessed the impact if the patients with “unknown” or missing ethnicity were included in the non-Hispanic group. This helped us quantify the magnitude of the potential bias due to the possible missingness of data on Hispanic ethnicity (Supplemental Tables 1 and 2 https://links.lww.com/AOSO/A672). We also assessed the impact of including 18.5% of patients with “unknown” or missing ethnicity in the Hispanic group and then the non-Hispanic group on the odds of mortality.

This study was exempt from review by our Institutional Review Board because NSQIP data contained no identifiable patient information.

## RESULTS

In total, 479,543 patients underwent cholecystectomy cases during the 15-year period studied. The mean age of the patients was 49.7 (standard deviations: 17.3) years. 12.5% of patients were Hispanic, 68.9% were non-Hispanic, and 18.5% were unknown or blank (Table 1). While 74.7% of Hispanic patients were female, 68.3% of non-Hispanic patients were female. 1.6% of Hispanic patients were Black, and 11.6% of non-Hispanic patients were Black. 11.4% and 19.0% of Hispanic and non-Hispanic patients smoked, respectively.

**TABLE 1.** Patient Characteristics and Outcomes by Ethnicity

		Ethnicity	
	Total	Hispanic	Non-Hispanic
	411,208	63,291	347,917
Mean age [SD]	49.7 [17.3]	43.3 [15.9]	50.9 [17.3]
Sex (%)			
Female	284,957	47,281 (74.7)	237,676 (68.3)
Male	126,251	16,010 (25.3)	110,241 (31.7)
Race (%)			
White	322,012	46,910 (74.1)	275,102 (79.1)
Black	41,239	860 (1.4)	40,379 (11.6)
Asian	14,824	163 (0.3)	14,661 (4.2)
American Indian/Alaska Native	3993	421 (0.7)	3752 (1.0)
Undetermined	29,140	14,937 (23.6)	14,203 (4.1)
Comorbidities (%)			
Diabetes	54,243	7766 (12.3)	46,477 (13.4)
Smoking	73,149	7185 (11.4)	65,964 (19.0)
Recent weight loss*	3537	332 (0.5)	3205 (0.9)
Mean body mass index [SD]	31.54 [10.9]	31.52 [20.9]	31.54 [7.8]
ASA classification†			
1 No disturbance	41,249	10,512 (16.6)	30,737 (8.8)
2 Mild disturbance	235,022	39,333 (62.1)	195,689 (56.2)
3 Severe disturbance	124,771	12,620 (19.9)	112,151 (32.2)
4 Life-threatening	10,086	822 (1.3)	9264 (2.7)
5 Moribund	80	4 (0.0)	76 (0.0)
Type of cholecystectomy (%)			
Emergency	40,822	9721 (15.4)	31,101 (8.9)
Urgent	31,163	4251 (6.7)	26,912 (7.7)
Inpatient elective	101,313	17,734 (28.0)	83,579 (24.0)
Outpatient	237,910	31,585 (49.9)	206,325 (59.3)
Surgical approach (%)			
Laparoscopic	387,032	60,641 (95.8)	326,391 (93.8)
Open	24,176	2650 (4.2)	21,526 (6.2)
30-Day mortality (%)			
Emergency	306	24 (0.0)	282 (0.1)
Urgent	167	8 (0.0)	159 (0.0)
Inpatient elective	664	41 (0.1)	623 (0.2)
Outpatient	170	8 (0.0)	162 (0.1)

* Recent weight loss is defined as more than 10% body weight loss in the last 6 months.

†ASA = American Society of Anesthesiologists.

Values in brackets for continuous variables mean age and body mass index are the standard deviations (SD).

N = 16,955 patients were excluded because they were missing at least one variable other than ethnicity.

N = 93,387 patients had missing ethnicity data and are excluded. Sensitivity analysis includes these patients.

**FIGURE 1.**
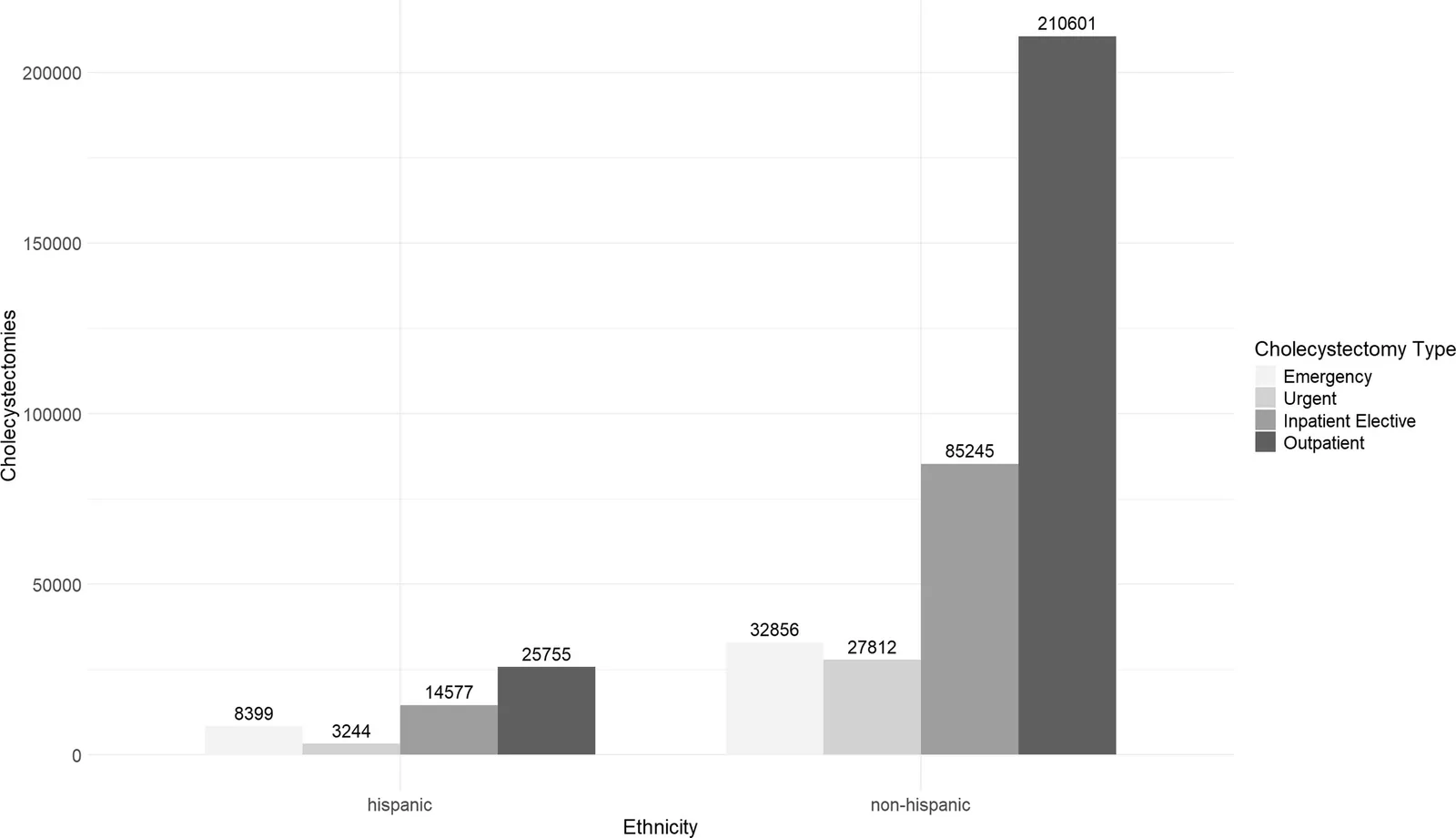
Frequency of type of cholecystectomy by ethnicity.

15.4% and 8.9% of Hispanic and non-Hispanic patients were emergent cholecystectomy cases, respectively. Within both Hispanic and non-Hispanic groups, outpatient cases were the most common, at 49.9% for Hispanic and 59.3% for non-Hispanic groups (Table 1 and Fig. 1). Unplanned cases were the least common (15.4% emergency and 6.7% urgent for Hispanic and 8.9% and 7.7% for non-Hispanic). 4.2% and 6.2% of Hispanic and non-Hispanic patients were open cholecystectomy cases, respectively.

**FIGURE 2.**
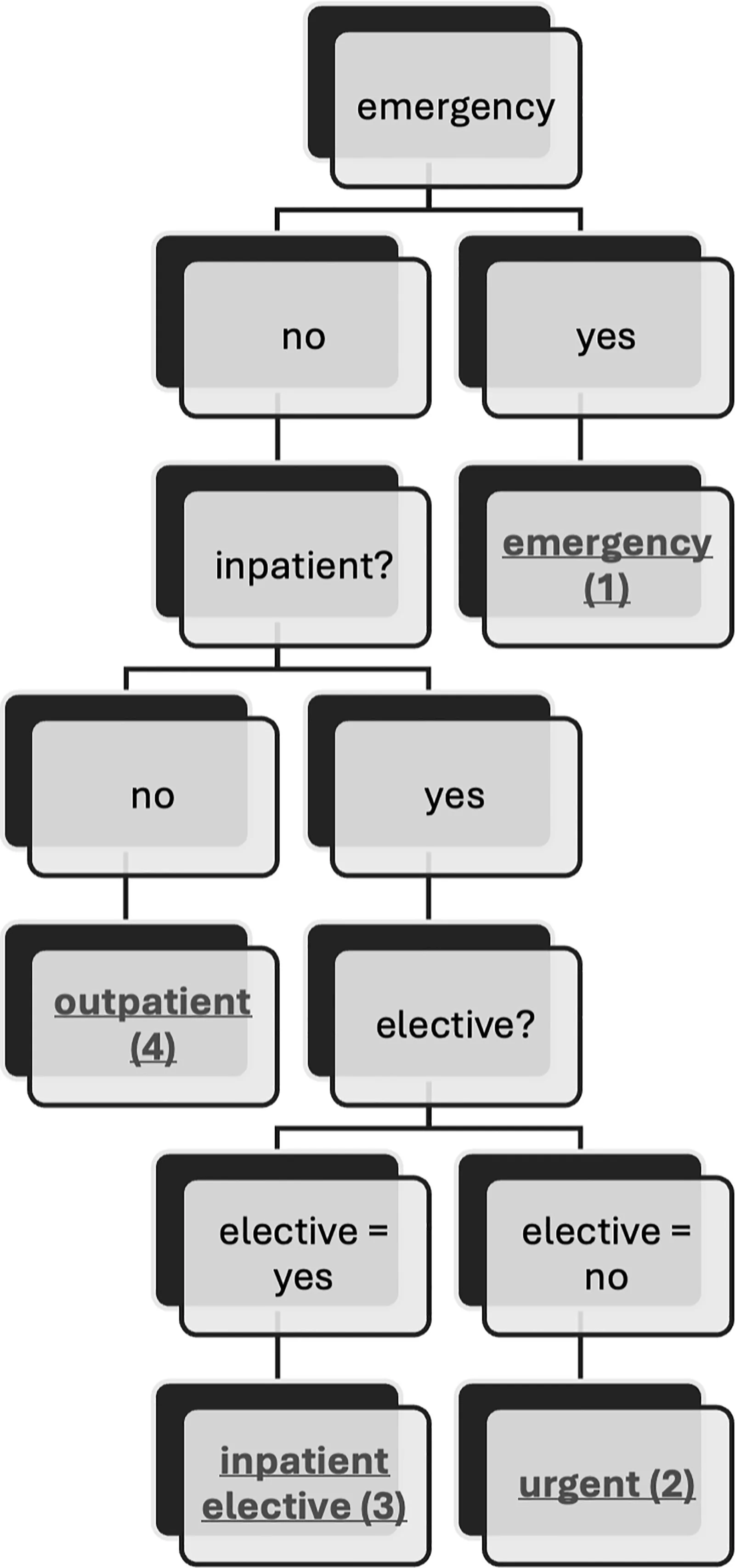
The primary outcome of the study was the type of cholecystectomy, separated into four levels: (1) emergency, (2) urgent [nonemergency-inpatient-nonelective], (3) inpatient elective [nonemergency-inpatient-elective], and (4) outpatient [nonemergency outpatient]. These definitions were determined using variables included in the NSQIP database, including emergency (yes/no), inpatient (yes/no), and election (yes/no).

Hispanic patients had increased odds of emergency compared to outpatient cholecystectomy versus non-Hispanic patients (adjusted OR [aOR] 2.32; 95% CI: 2.255–2.385) (Table 2). In addition to our primary analysis of emergency compared with outpatient cholecystectomy cases, Hispanic patients had increased odds of unplanned cholecystectomies (emergency or urgent) as compared with planned cholecystectomies (inpatient elective or outpatient) [aOR: 1.592; CI: 1.556, 1.628) (Table 2). Upon cumulative regression, Hispanic patients also had 89% increased odds of emergency (1) versus nonemergency (2, 3, 4) cholecystectomy (aOR: 1.885; CI: 1.838–1.933) (Table 3). Additionally, Hispanic patients had 76% increased odds of inpatient (1, 2, 3) versus outpatient (4) cholecystectomy (aOR: 1.756; CI: 1.725–1.787) (Table 3).

**TABLE 2.** The Odds of Type of Cholecystectomy by Ethnicity

Outcomes	Unadjusted Odds Ratio (uOR)	95% CI for uOR (Lower, Upper)	Adjusted Odds Ratio (aOR)*	95% CI for OR (Lower, Upper)
Emergency (1) vs outpatient (4)				
Hispanic	2.042	(1.990, 2.095)	2.319	(2.255, 2.385)
Non-Hispanic	Reference		Reference	
Unplanned (1 + 2) vs Planned (3 + 4)				
Hispanic	1.416	(1.387, 1.445)	1.592	(1.556,1.628)
Non-Hispanic	Reference		Reference	

* Logistic regression model with ethnicity as an independent variable, adjusted for sex, age, race, recent weight loss, body mass index, smoking, diabetes, and ASA classification.

Gray shaded region indicates secondary analysis.

1 = emergency; 2 = urgent; 3 = inpatient elective; 4 = outpatient

**TABLE 3.** The Odds of Type of Cholecystectomy by Ethnicity, Reflecting Ordered Nature

Outcomes	Unadjusted Odds Ratio (uOR)	95% CI for uOR (lower, upper)	Adjusted Odds Ratio (aOR)*	95% CI for aOR (lower, upper)
Emergency (1) vs Outpatient (4)				
Hispanic	2.042	(1.990, 2.095)	2.319	(2.255, 2.385)
Non-Hispanic	Reference		Reference	
Emergency (1) vs Nonemergency (2, 3, 4)				
Hispanic	1.849	(1.804, 1.894)	1.885	(1.838, 1.933)
Non-Hispanic	Reference		Reference	
Unplanned (1 and 2) vs Planned (3 and 4)				
Hispanic	1.416	(1.387, 1.445)	1.592	(1.556, 1.628)
Non-Hispanic	Reference		Reference	
Inpatient (1, 2, 3) vs Outpatient (4)				
Hispanic	1.463	(1.438, 1.488)	1.756	(1.725, 1.787)
Non-Hispanic	Reference		Reference	

* Polynomial ordinal regression model with ethnicity as the independent variable, adjusted for sex, age, race, recent weight loss, body mass index, smoking, diabetes, and ASA classification.

1= emergency; 2=urgent; 3=inpatient elective; 4=outpatient

Hispanic patients who underwent unplanned cholecystectomies had 79% decreased odds of mortality compared to non-Hispanic patients (aOR: 0.211; CI: 0.194, 0.229) (Table 4). In addition, Hispanic patients had 65% decreased odds of mortality for planned surgery (aOR: 0.354; CI: 0.329, 0.381) (Table 4) compared with non-Hispanic patients.

**TABLE 4.** Odds of 30-Day Mortality of Unplanned and Planned Cholecystectomy by Ethnicity

Type of Surgery	Unadjusted Odds Ratio (uOR)	95% CI for uOR (Lower, Upper)	Adjusted Odds Ratio (aOR)*	95% CI for aOR (Lower, Upper)
Unplanned (1 + 2)				
Hispanic	0.300	(0.209, 0.429)	0.211	(0.194, 0.229)
Non-Hispanic	Reference		Reference	
Planned (3 + 4)				
Hispanic	0.366	(0.274, 0.489)	0.354	(0.329, 0.381)
Non-Hispanic	Reference		Reference	

* Logistic regression model with ethnicity as an independent variable, adjusted for sex, age, race, recent weight loss, body mass index, smoking, diabetes, ASA classification, and open vs. laparoscopic surgery.

1 = emergency; 2 = urgent; 3 = inpatient elective; 4 = outpatient

Regarding cholecystectomy case type, our sensitivity analysis assigning the unknowns to Hispanic and non-Hispanic groups separately had similarly increased odds to our main analyses that excluded the unknowns (Supplemental Table 1, https://links.lww.com/AOSO/A672).

For 30-day mortality of unplanned cholecystectomies, there was an increase in the OR between the primary analysis (aOR: 0.211; CI: 0.194, 0.229) and the sensitivity analysis with unknowns assigned to the Hispanic group (aOR: 0.534; CI: 0.431, 0.663; Supplemental Table 2, https://links.lww.com/AOSO/A672). Similarly, there was an increase in the OR in the sensitivity analysis with the unknowns assigned to the non-Hispanic group (aOR: 0.422; CI: 0.344, 0.518; Supplemental Table 2, https://links.lww.com/AOSO/A672).

For 30-day mortality of planned cholecystectomies, there was a decrease in the OR between the primary analysis (aOR: 0.354; CI: 0.329, 0.381) and the sensitivity analysis with unknowns assigned to the non-Hispanic group (aOR: 0.137; CI: 0.119–0.158; Supplemental Table 2, https://links.lww.com/AOSO/A672). The sensitivity analysis with unknowns assigned to the Hispanic group made the effect disappear (aOR: 1.12; CI: 0.968–1.29; Supplemental Table 2, https://links.lww.com/AOSO/A672).

## DISCUSSION

This study of Hispanic patients, types of cholecystectomy cases, and postoperative case mortality had two main findings. First, we found that compared with non-Hispanic patients, Hispanic patients were more than twice as likely to undergo an emergency versus outpatient cholecystectomy. Next, Hispanic patients had decreased mortality for both unplanned and planned cholecystectomies compared with non-Hispanic patients.

Our analysis showed that Hispanic patients were more likely than non-Hispanic patients to have emergency compared with outpatient cholecystectomy, which supports previous findings of underrepresented minority patients having fewer elective and ambulatory procedures.^16^ However, the present study adds to the literature by further specifying that in addition to more frequently undergoing inpatient cholecystectomy, these inpatient surgeries are more often designated emergent and urgent in these groups. Importantly, our study adds more specific information on Hispanic patients using a representative sample of 479,543 cholecystectomies over a 15-year period and uniquely contextualizes all possible types of cholecystectomies for a comprehensive look at surgical treatment of gallstone disease. We investigated the Hispanic population and their experiences with all 4 cholecystectomies: emergency, urgent, inpatient elective, and outpatient. Hispanic patients possibly encounter disparities in timely diagnosis of their gallstone disease at each level of disease severity. In the present study, Hispanic patients underwent significantly more unplanned cholecystectomies as compared with non-Hispanic patients. Planned, outpatient cholecystectomy has been demonstrated as a less costly alternative to inpatient cholecystectomy and is associated with fewer unplanned admissions and greater patient satisfaction.^29–31^ Thus, it is important to understand the etiology of Hispanic patients being less likely than non-Hispanic patients to undergo timely, safe, and affordable patient-centric outpatient cholecystectomies.

Whether biological, medical, or sociocultural etiologies contribute to these findings cannot be determined using these case–control data. Due to the known increased biological risks of gallstone disease in the Hispanic population, surgeons may have performed more acute surgery for gallstone disease in Hispanic patients.^32^ Alternatively, concerns of language proficiency or health literacy in Hispanic patients may prompt surgeons to declare cases as emergent or urgent (regardless of disease severity) to avoid perceived barriers to scheduling outpatient cholecystectomy.^33–35^ Beyond influencing surgeon decision-making at the point of care, language discordance may also contribute to delays in initial presentation for clinical care, resulting in more advanced disease at the time of surgical evaluation and a higher likelihood of emergent intervention; this has been associated with worse perioperative and surgical outcomes across a range of procedures.^36^

We found that Hispanic patients have decreased postoperative mortality as compared with non-Hispanic patients for unplanned cholecystectomies, which we hypothesize can be explained by biological or social variables. It is possible that a higher proportion of Hispanic patients with clinically nonemergent disease are being treated with unplanned cholecystectomy as a workaround. It is also possible that Hispanic patients could have a health factor that protects them from mortality during or after more acute forms of surgery. This so-called Hispanic paradox might highlight Hispanic patients’ physiology, which may include a better fit for higher-risk cholecystectomy owing to less physiological debt upon entering surgery and completing phases of postoperative recovery to achieve a more favorable outcome.^37^

Although our study has many strengths, including the generalizability of the nationally representative surgical dataset, it has some important limitations. Although NSQIP aggregates data from a large number of hospitals with good regional coverage, there is an annual subscription fee of approximately $80,000 for hospitals to participate.^38^ Thus, if patients at safety-net hospitals are more likely to be underrepresented minorities and costs prohibit these hospitals from participating, this could lead to a selection bias against certain patient ethnicities and socioeconomic statuses.^39^ Additionally, if NSQIP hospitals are contributing inpatient and outpatient case data differently to the database, this can bias our measure of effect. Furthermore, NSQIP hospitals may have enhanced systematic infrastructure and processes that decrease the postoperative mortality of Hispanic patients more than hospitals that do not participate.

Residual confounding is inherent to any observational study due to unmeasured or unobserved variables. As this study utilizes NSQIP electronic medical record data from participating hospitals, we are particularly limited by which potential confounders we can include in our regression analyses. For example, we were unable to account for insurance, state of residence, or primary language, not to mention other potential biological variables in the causal pathway, such as serum low-density lipoprotein cholesterol level, gut microbiome, medication use, and gastrointestinal motility risk contained in other population-level longitudinal electronic health records data.^40,41^ However, given previous findings that the risk of emergency cholecystectomy and case fatality in the Latino population increases with Medicaid expansion here in the United States, we expect the disparities between emergency and ambulatory surgeries to be even greater with public insurance coverage.^10^ It is possible the absence of an insurance variable, thus, would bias mortality results toward the null. Additionally, the low death numbers (especially in the Hispanic, unplanned cholecystectomy group) in our patient sample may destabilize our mortality regression analysis based on the number of variables in our adjusted model. As such, these aORs should be interpreted with caution.

An important consideration is that 18.5% of patients had unknown or missing Hispanic ethnicity data, which could potentially increase the misclassification of exposure. However, our sensitivity analyses of cholecystectomy type demonstrated that the effect remains the same when the unknowns are assigned to either the Hispanic or non-Hispanic groups and that our findings are not biased by the missingness of ethnicity data. In contrast, our sensitivity analyses for mortality showed that the possible missingness of ethnicity data could have biased the results. Specifically, assigning the unknowns to non-Hispanic or Hispanic in the unplanned settings decreased the Hispanic Paradox, albeit still present. However, while assigning the unknowns to non-Hispanic strengthened the Hispanic Paradox in the planned settings, assigning the unknowns to Hispanic in the planned settings eliminated this effect seen in the primary analyses of mortality. Thus, if there is bias in assigning patient ethnicity as unknown, this could challenge the Hispanic paradox.^28^ Another possible explanation for this alteration of effect is random error. Additionally, although our mortality difference is statistically significant, and the number of deaths between patient groups is relevant, the low number of deaths within patient groups might reduce the clinical significance of our reported OR and thus should be interpreted with caution.

Bias in our study could also result from the potential misclassification of the outcome with the operationalization of the NSQIP variables. Previous studies focusing on one comparison, such as ambulatory versus inpatient, urgent versus elective, or unplanned versus elective, made their own assumptions in the definition.^9,16,42^ We assumed any case that was labeled “emergency” in NSQIP was a true clinical emergency, although it is possible some cases are “booked” as an emergency to expedite a case for many practical, patient-centered reasons, working with the limitations of the hospital system, for example, a patient’s second presentation or a language barrier or immigrant status that would impact their ability to follow-up and successfully schedule an outpatient case. Our assumption biases the results away from the null.^35,43^ The random error was extremely low in our study; that is, the CIs were very narrow.

In conclusion, while previous studies have shown that underrepresented groups are more likely to have had “emergency” or “inpatient” or “urgent” surgery, we find that regardless of how the more severe, less patient-centered form of cholecystectomy is defined, Hispanic patients are more likely to have the riskier form of cholecystectomies than the safer, more patient-centered form. Hispanic patients have greater odds of emergency than outpatient, emergency than nonemergency, unplanned than planned, and inpatient than outpatient cholecystectomy. Ethnic disparity holds regardless of how Hispanic patients are treated with cholecystectomy. Furthermore, despite Hispanic patients having a lower likelihood of dying than non-Hispanic patients, this lower likelihood is less pronounced in the planned setting, albeit biased by possible misclassification of Hispanic ethnicity. There is potential to better understand the mechanisms at play attributed to Hispanic patients having more emergency and fewer nonemergency cholecystectomies than non-Hispanic patients, as well as why they receive less benefit from planned cholecystectomy. Our follow-up study includes an interview of patients who have experienced emergency compared to elective cholecystectomies to further understand (1) the patient-reported physical symptoms of gallstone disease, (2) patient interpretation of gallstone disease symptoms, and (3) patient utilization of their interpersonal social networks during symptoms of gallstone disease that our present research question and NSQIP dataset study do not address.^44^

## Supplementary Material


