# Supplementary material for: The Association Between Hispanic Ethnicity, Cholecystectomy, and Postoperative Mortality
Source: Ann Surg Open. 2026 Sep 10;7(3):e709. doi: 10.1097/AS9.0000000000000709 (PMC13596973; doi:10.1097/AS9.0000000000000709)
Supplement: Supplementary file 1 [file as9-7-e709-s001.pdf]

Appendix 1

Category	Code	Description
CPT Procedure Codes	CPT-4: 47562	Laparoscopic cholecystectomy
	CPT-4: 47563	Laparoscopic cholecystectomy with cholangiography
	CPT-4: 47564	Laparoscopic cholecystectomy with exploration of common duct
	CPT-4: 47600	Cholecystectomy (open)
	CPT-4: 47605	Cholecystectomy with cholangiography (open)
	CPT-4: 47610	Cholecystectomy with exploration of common duct (open)
	CPT-4: 47612	Cholecystectomy with exploration of common duct and choledochoenterostomy (open)
	CPT-4: 47620	Cholecystectomy with exploration of common duct and transduodenal sphincterotomy/sphincteroplasty (open)
ICD-10 Diagnosis Codes	ICD-10: K80.x	Cholelithiasis (gallstones), with/without cholecystitis, with/without obstruction (all subcodes)
	ICD-10: K81.x	Cholecystitis (all subcodes)
	ICD-10: K82.x	Other diseases of gallbladder, including gallbladder dysfunction (all subcodes)
	ICD-10: K85.1x	Biliary acute pancreatitis
	ICD-10: K56.3	Gallstone ileus
ICD-9 Diagnosis Codes	ICD-9: 574.x	Cholelithiasis, all subtypes (with/without cholecystitis, with/without obstruction)
	ICD-9: 575.x	Other disorders of gallbladder (cholecystitis, hydrops, perforation, fistula, cholesterolosis, dyskinesia)
	ICD-9: 576.x	Other disorders of biliary tract (postcholecystectomy syndrome, biliary fistula, spasm of sphincter of Oddi)

ICD-9, ICD-10, and CPT Coding Algorithm.
