# Supplementary material for: The Association Between Hispanic Ethnicity, Cholecystectomy, and Postoperative Mortality
Source: Ann Surg Open. 2026 Sep 10;7(3):e709. doi: 10.1097/AS9.0000000000000709 (PMC13596973; doi:10.1097/AS9.0000000000000709)
Supplement: Supplementary file 2 [file as9-7-e709-s002.pdf]

Supplemental Tables

Supplemental Table I.
Odds of Type of Cholecystectomy by Ethnicity when Assigning Unknown Ethnicity

Outcomes	Unadjusted Odds Ratio (uOR)	95% CI for uOR (lower, upper)	Adjusted Odds Ratio (aOR)*	95% CI for aOR (lower, upper)
**Emergency (1) vs Outpatient (4)**				
Non-Hispanic	Reference		Reference	
Unknowns assigned to Hispanic group	1.649	(1.617, 1.681)	1.887	(1.839, 1.936)
Unknowns assigned to Non-Hispanic group	1.881	(1.835, 1.928)	2.167	(2.111, 2.224)
Primary Analysis	2.042	(1.990, 2.095)	2.319	(2.255, 2.385)
**Emergency (1) vs Nonemergency (2,3,4)**				
Non-Hispanic	Reference		Reference	
Unknowns assigned to Hispanic group	1.586	(1.557, 1.616)	1.606	(1.576, 1.637)
Unknowns assigned to Non-Hispanic group	1.703	(1.663, 1.744)	1.746	(1.705, 1.788)
Primary Analysis	1.849	(1.804, 1.894)	1.885	(1.838, 1.933)
**Unplanned (1+2) vs Planned (3+4)**				
Non-Hispanic	Reference		Reference	
Unknowns assigned to Hispanic group	1.252	(1.238, 1.271)	1.438	(1.410, 1.468)
Unknowns assigned to Non-Hispanic group	1.374	(1.347, 1.402)	1.551	(1.519, 1.584)
Primary Analysis	1.416	(1.387, 1.445)	1.592	(1.556, 1.628)
**Inpatient (1,2,3) vs Outpatient (4)**				
Non-Hispanic	Reference		Reference	
Unknowns assigned to Hispanic group	1.232	(1.217, 1.247)	1.376	(1.359, 1.393)
Unknowns assigned to Non-Hispanic group	1.435	(1.411, 1.459)	1.694	(1.665, 1.723)
Primary Analysis	1.463	(1.438, 1.488)	1.756	(1.725, 1.787)

*Sensitivity analyses of regression models that assign the 93,387 patients with unknown ethnicity status to either the non-Hispanic group or Hispanic group to assess possible biases due to missing Hispanic data.
1= emergency; 2=urgent; 3=inpatient elective; 4=outpatient

Supplemental Table II.
Odds of 30-Day Mortality of Unplanned and Planned Cholecystectomy when Assigning Unknown Ethnicity

Type of Surgery	Unadjusted Odds Ratio (uOR)	95% CI for uOR (lower, upper)	Adjusted Odds Ratio (aOR)[a]	95% CI for aOR (lower, upper)
**Unplanned (1+2)**				
Non-Hispanic	Reference		Reference	
Unknowns in Hispanic group	0.484	(0.395, 0.594)	0.534	(0.431, 0.663)
Unknowns in Non-Hispanic Group	0.328	(0.229, 0.468)	0.422	(0.344, .518)
Primary Analysis	0.300	(0.209, 0.429)	0.211	(0.194, 0.229)
**Planned (3+4)**				
Non-Hispanic	Reference		Reference	
Unknowns in Hispanic group	0.562	(0.480, 0.659)	1.12	(0.968, 1.29)
Unknowns in Non-Hispanic Group	0.392	(0.294, 0.522)	0.137	(0.119, 0.158)
Primary Analysis	0.366	(0.274, 0.489)	0.354	(0.329, 0.381)

[*]Sensitivity analyses of mortality analysis that assign the 93,387 patients with unknown ethnicity status to either the non-Hispanic group or Hispanic group to assess possible biases due to missing Hispanic data.
1= emergency; 2=urgent; 3=inpatient elective; 4=outpatient
